# Thalidomide inhibits inflammation and angiogenesis in tumor 4T1 growth in mice

**DOI:** 10.1186/1753-6561-7-S2-P18

**Published:** 2013-04-04

**Authors:** Cristina M Souza, Cecília B Campos, Ana CA Silva, Míriam P Lopes, Mônica AND Ferreira, Silvia P Andrade, Geovanni D Cassali

**Affiliations:** 1 Department of General Pathology, Laboratory of Comparative Pathology, Biological Sciences Institute, Federal University of Minas Gerais, Belo Horizonte, Minas Gerais, Brazil; 2 Department of Physiology and Biophysics, Laboratory of Angiogenesis, Biological Sciences Institute, Federal University of Minas Gerais, Belo Horizonte, Minas Gerais, Brazil; 3 Department of Pharmacology, Laboratory of Antitumor Substances Biological Sciences Institute, Federal University of Minas Gerais, Belo Horizonte, Minas Gerais, Brazil

## Background

Thalidomide has proven to exert anti-inflammatory, anti-proliferative and anti-angiogenic activities in both neoplastic and non-neoplastic conditions. We investigated the effects of this compound on blood vessel formation, inflammatory cell recruitment/activation and cytokine production of 4T1 mammary tumor in mice.

## Materials and methods

4T1 cells were injected subcutaneously into Balb/c mice. After tumor engraftment (5 days), thalidomide (150 mg/kg) was administered to the treated group for 7 days. Tumors of control and treated groups were sized regularly, removed 12 days after inoculation and processed for biochemical parameters to assess neovascularization and inflammation.

## Results

Daily oral dose of thalidomide was able to reduce in 46% the tumor volume. Assessment of tumor vascularization revealed a significant decrease in blood vessels formation by thalidomide. The levels of two cytokines, VEGF and TNF-α were decreased in tumor samples of thalidomide-treated group compared with the control group. Accumulation of neutrophils or macrophages in the 4T1 tumor measured by the activities of inflammatory enzymes, MPO for neutrophils and NAG for macrophages, was inhibited by the treatment.

## Conclusions

By targeting key components of 4T1 tumor simultaneously, thalidomide was effective in attenuating tumor growth. This approach, suppression of inflammation and angiogenesis may provide further insights for both prevention and treatment of cancer.

## Financial support

FAPEMIG, CNPq and CAPES.

